# Hand luggage in the train toilet

**DOI:** 10.3233/WOR-182689

**Published:** 2018-04-06

**Authors:** M. Loth, J.F.M. Molenbroek, D.J. van Eijk

**Affiliations:** Faculty of Industrial Design Engineering, Delft University of Technology, Delft, The Netherlands

**Keywords:** Train toilet, hand luggage, coat, bags, storage and observational research

## Abstract

**BACKGROUND:**

The train toilet can form a barrier for those wishing to travel by train as it is perceived as being dirty, and therefore its use as being unpleasant. In addition, Dutch train toilet users have the additional issue of storing their hand luggage in the toilet’s confined space

**OBJECTIVE:**

In this article, we examine the issue of Dutch travelers with hand luggage in relation to their use of train toilets. We investigate the type of hand luggage train travelers have with them and lastly, we study what travelers do with their hand luggage when using the toilet.

**METHODS:**

As part of an overarching study, we asked two specific questions on what travelers do with their hand luggage in a train toilet environment, followed by 22 observations from observational research.

**RESULTS:**

In the questionnaire, train travelers reported that bringing hand luggage into the train toilet is a problem because of the lack of storage space, and their fear of losing their seat. From the observational research, we noted that the participants mainly held their hand luggage on their bodies, and to a lesser extent, they placed it on the floor of the train toilet itself. None of the 22 participants used the hook to hang up their bag and/ or their coat.

**CONCLUSIONS:**

Travelers need a facility in the train toilet to store their hand luggage. Women have a stronger need for this than men, as they almost always carry an item with them. In addition, they use the toilet in hovering position or seated, with their backs to the wall, so they have limited space to store hand luggage on their backs or shoulders as men do. Most participants kept their hand luggage at a distance from the bowl, and the majority kept it off the floor (14 of the 22) because they were aware of the hygiene. The positioning of the coat/luggage hook at 1840 mm above the floor was considered to be too high, out of people’s comfort area.

## 1 Introduction

Every day, 1.1 million customers travel on the Dutch National Railway (Nederlandse Spoorwegen-NS) trains [1]. An important characteristic of most commuters is that they have some form of (hand) luggage with them; they carry “more than [just] a rolled-up” newspaper [2 px], or mobile phone.

In the Dutch train compartment itself, different storage places are available, including hooks to hang up a coat, overhead luggage racks, and spaces behind the seat for luggage storage. In all NS train toilets however, only a hook is available for hanging up a bag and/or coat. That leads us to the focus of this article in which we report on what travellers do with their hand luggage when using the train toilet.

Firstly, let us define what we mean by hand luggage. For this paper, we consider it to be small luggage that can be easily handled and stored on the body, with an estimated weight of less than 5 kg, including coats and excluding suitcases and trolleys. We define a handbag as a small, handheld bag with a small strap. A (shoulder) bag can be used by both men and women, is larger than a handbag, and has a strap attached that can be slung over the shoulder. The largest type of hand luggage is a weekend/sports bag; a large bag attached with a large strap that can be slung over the shoulder (own observation). Finally, some travellers take medical equipment with them, such as colostomy equipment, catheters, walkers and wheelchairs [2]. In this study, we only observed one participant fitted with a stoma; in a separate mock-up study as part of the PhD thesis (to be published), we observed people with catheters, walkers and wheelchairs.

This article forms part of a PhD project ‘Hygiene in the train toilet’, a cooperation between Delft University of Technology (DUT) and NS. It has been shown that 83% of Dutch train travellers take all kinds of effort to avoid using the train toilet due to the poor hygiene [3]. People even avoid travelling by train (findings from a questionnaire as part of the PhD thesis, to be published at a later date, see section 2.1) or choose to stay at home for the same reasons [4].

To reverse this undesirable situation, NL Agency, formerly known as SenterNovem, provided start-up funding for the PhD thesis project, to research the use of train toilets and to develop a ‘Hygienic Train Toilet’, as this partially funded project from Dutch Railways and SenterNovem has been named. The overall goal of this PhD project is to improve train toilet hygiene through ergonomic design research in order to provide travellers with more comfort, thereby removing a possible barrier to travelling by train [5, 6].

The main elements of the PhD study (in progress) are an extensive questionnaire with train travellers and a comprehensive observational research study of their use of toilets and hygiene. The aim of the questionnaire was to characterise train travellers and to identify their needs regarding the use of the toilet. We included two questions on what travellers carry with them as hand luggage.

**Fig.1:**
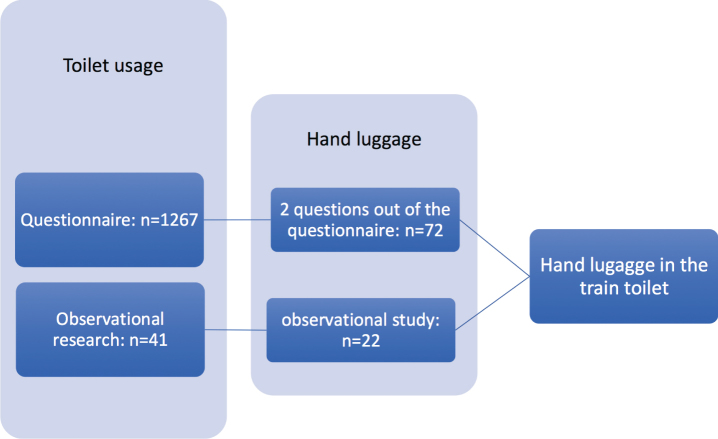
Outline method.

In the observational research, conducted in moving NS trains, we recorded how travellers use the train toilet to gain an understanding of how toilets become so unhygienic, and what they do with their coats and hand luggage. In addition, we looked specifically how 22 participants used the facilities for storing luggage in the toilets (Fig. 1, section 2)

## 2 Method

The questionnaire and the observational research are complementary; the extensive questionnaire served as an introduction to the observational research to determine which aspects we should focus on.

From the two questions on hand luggage used in the questionnaire, we learned that hand luggage is an issue for travelers using the train toilet. In the observational research, we focused on the aspects of carrying hand luggage into the train toilet, and what travelers did with it when using the toilet, see Fig. 1.

### 2.1 Questionnaire

As part of the PhD project ‘Hygiene in the train toilet’, we developed an extensive questionnaire with 75 mainly closed multiple-choice questions to determine travelers’ needs and use of the train toilet. The survey was conducted in February 2010.

We approached 3960 panelists from the NS panel (http://nspanel.nl) by email. Of these, 1267 panelists completed the survey; a response of 32%. The 1267 respondents were representative for Dutch train travelers, but not for the Dutch population. Details of the respondents’ background information like gender, age, travel frequency, and length of train travel will be published in depth at a later date.

In this paper, we only consider the aspect of gender related to the demand of luggage storage.

Two questions specifically related to the issues of dealing with hand luggage and personal belongings in the train toilet were completed by 72 respondents, all of whom were Dutch train travelers.

1. I do not want to/cannot take my luggage into the toilet:
1.1 because of a lack of storage.
1.2 if I take my belongings with me I lose my seat.

To gain insight into the types of hand luggage that travelers carry with them on their trip, they responded to the next open question:

2. Can you give a description of the hand luggage/personal belongings?

### 2.2 Observational research

We video-recorded 41 participants visiting a train toilet in a moving train. These observations were conducted in the context of toilet usage, however 22 recordings were also valid for their handling of hand luggage in the train toilet. In this article, we describe how these 22 participants dealt with hand luggage such as coats and different kinds of bags in the train toilet, in a near-realistic setting.

The participants gave permission to be videotaped by signing the informed consent forms to guarantee their privacy. Furthermore, outline figures were made of their performances to make them completely unrecognizable in publications.

**Fig.2:**
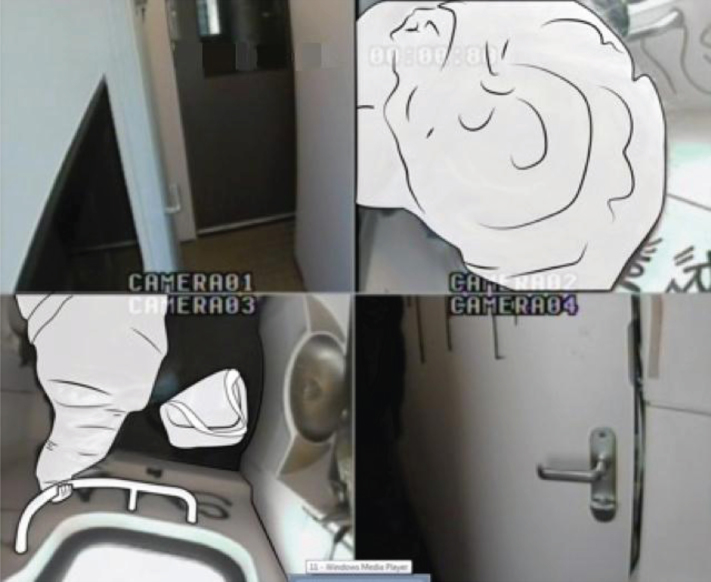
Four camera viewpoints, and bag in the small train toilet.

**Fig.3:**
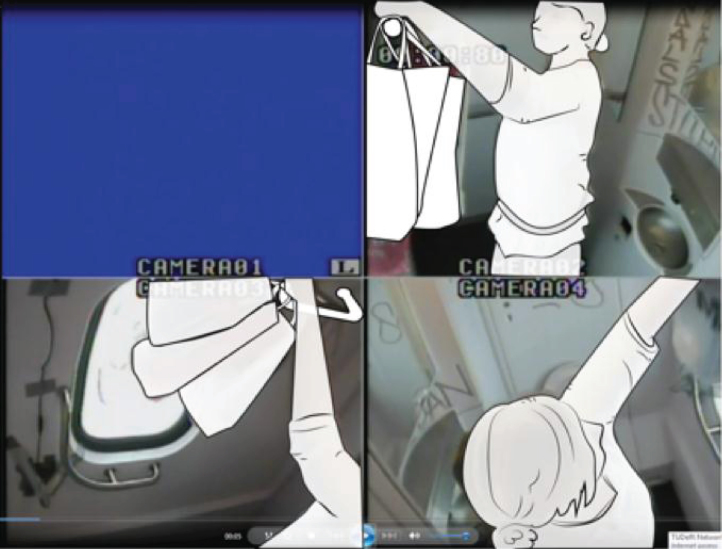
One camera was disconnected, and plastic shopping bags in large train toilet.

In the toilets, we installed four cameras to ensure that four viewpoints were visible on the computer screen (Figs. 2 and 3). Further, the research group studying a specific group of participants (Nr. 15-Nr.25), (Fig. 14, section 3.2) disconnected one camera for female participants in order to ensure a less clear view of her private body part (Fig. 3).

The recordings enabled us to review the observations coded by the author using a special observation program, Observer XT [7].

In this article, we pooled the 41 observations and noted 22 observations where participants dealt with the following types of (hand) luggage in the train toilet: plastic shopping bags, weekend bags, backpacks and coats. The research group studying participants Nr.15-Nr.25 focused on hand luggage and encouraged them to take hand luggage into the train toilet. These participants could choose from plastic shopping bags or a weekend bag in addition to their own hand luggage. In 11 observations, participants already took their own backpack, coat and/or bag with them to the toilet, although this had not been specifically requested. We conducted nine observational studies in the small train toilet, and 13 in the large one; see Fig. 14; table hand luggage, section 3.2.

The aim of this observational study was to explore where travelers leave their hand luggage in a train toilet.

#### 2.2.1 Participants

The research team recruited the 41 participants from their network, and informed them about the aim of the research: to study how people use train toilets in a realistic context. The team explained that they would be videotaped while using the train toilet, and they were asked to sign an informed consent form to guarantee their privacy. Further they were rewarded with a travel card for one day of unlimited first-class train travel; a value of about 80 Euro.

We recruited a mixed group of participants in order to represent the natural diversity of train travelers: age-range (24–57 years), gender (6 females and 16 males), physical capability (one used crutches, and one a stoma), profession (younger ones were students, and the older were workers). Their intensity of train travel varied.

There was only one precondition: they all needed to have had previous experiences with train toilets.

In our sample, many young, “able-bodied” male [8 p12] students were involved as Dutch students frequently travel by train in the Netherlands; they receive a free public transport card for the duration of their study [9].

Fig. 14, section 3.2; table hand luggage in the train toilet, presents details of hand luggage as well as the information on age group, and gender.

#### 2.2.2 Trains, toilets, and hook

For the research to take place, the NS provided a standard train used in daily service: the Double Decker Intercity VIRM [10]. This train has a small train toilet located in the front compartment of the train (Fig. 2), with a large toilet for disabled users, at the rear (Fig. 3).

**Fig.4:**
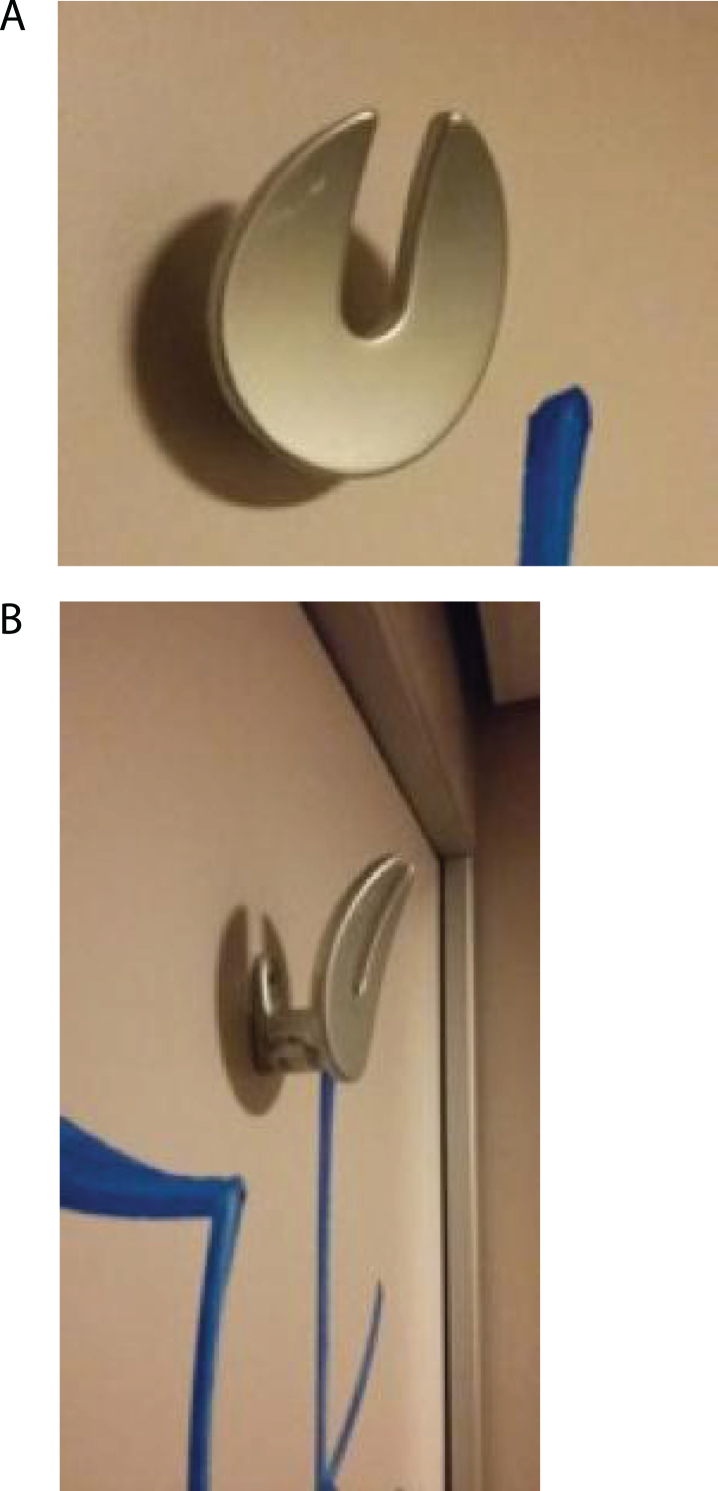
(A, B) Current hook in the train toilet.

**Fig.5:**
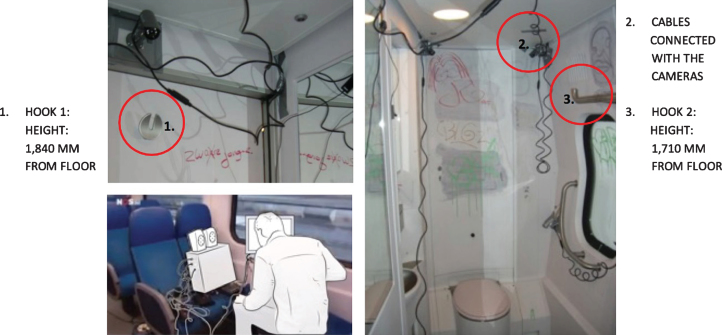
Impression of the context and installation of cameras.

The toilets have a specially- designed hook, the same as in the compartments of the train, inside the train door at the height of 1840 mm from the floor, which, for example, can be used to hang a coat, Fig. 4A, B. It can carry a maximum weight of 30 kg [11]. For the observations, we equipped these toilets with cameras and observation equipment (Fig. 5).

#### 2.2.3 Procedure

On Tuesday morning, 9 March 2010, participants and researchers met on a platform at The Hague Hollands Spoor station (H.S.) and stepped into the double-decker train to travel between the Hague-Amsterdam and back, a journey of approximately 120 minutes. Two weeks later, on 23 March 2010, a second train was scheduled for a shorter journey between the Hague and Leiden and back; approximately 50 minutes. The procedure used in both research trains was similar. In our experiments, each train was exclusively occupied by people involved in the research projects including research students, participants and supporting staff.

The lead researcher welcomed everyone in the upper part of the train, thanked the participants and then introduced the research team. The procedure was explained, and we emphasized that they could stop if they felt uncomfortable, and /or disallow the use of any observations.

Drinks and snacks were available in the top level of the double-decker which has been arranged to create a comfortable environment and to stimulate the participants to use the toilet. The research students gave as few instructions as possible in order to create as ‘normal’ a train journey situation as possible. The participants were able to use the toilet as and when they needed. Figure 5 shows the set up. The research students did not interfere with the participants while they were using the toilet. Following the toilet visit, the participants returned to their seat. Only members of the research team were authorized to view the private images.

In this article, we report on the handling of hand luggage in the train toilet as part of an ergonomics design PhD project investigating travelers’ use of train’s toilets.

There is almost no literature to be found on hand luggage handling in relation to public toilets; any literature found mainly referred to design solutions for storage, like luggage zone and shelves [2 p209, 12 p36, 13 p283].

## 3 Results

### 3.1 Questionnaire

The responses to the luggage questions was relatively low: 72/1267 = 6% 

**Table:** 

1.	I do not want to/cannot take my luggage into	*n* = 72
the toilet:
1.1	because storage lacks:	*n* = 28
1.2	If I take my belongings with me I lose my seat:	*n* = 22
1.3	both reasons (1.1 and 1.2):	*n* = 22
2.	Can you give a description of your luggage?	*n* = 72

Thus, approximately 1/3 of the respondents (*n* = 28) answered that there is no appropriate place to store their luggage, approximately 1/3 (*n* = 22) reported they were afraid of losing their seat, and approximately 1/3 (*n* = 22) gave both reasons.

**Fig.6:**
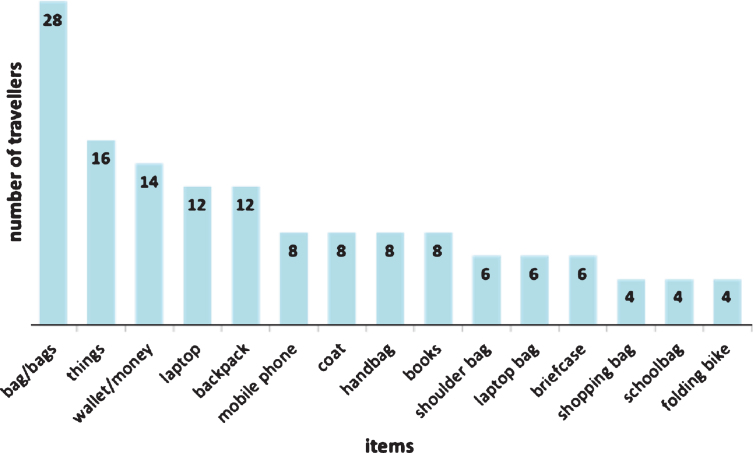
Description of the hand luggage of Dutch train travelers.

The respondents described their hand luggage as noted in Fig. 6 where n is the number of people that answered the open question describing their luggage: The top 5 items of hand luggage travelers take along on their journey are: (1) bag (s), (2) ‘things’, (3), wallet/money, shared (4): laptop and backpack, and shared (5): mobile phone, coat, handbag, and books.

‘Things’ are the second most mentioned luggage items. However, the respondents did not explain exactly what they meant by this. We deduct that this represents the ‘small loose items’ that passengers carry with them, such as a phone, keys, and wallets; personal items that people “like to have stored close by” or that they carry “in their own pockets” [14 p659, p662].

Respondents with suitcases and trolleys were not included in the observational study.

### 3.2 Results observational research

#### 3.2.1 Places where the participants kept their hand luggage

The observations showed that none of the participants used the hook 1 located inside the toilet door at the height of 1840 mm from the floor, (Figs. 4A, B, and 5), however, they placed their hand luggage in the following places:

(1) on their body (Fig. 8A, B, C), (2) on the ground (Fig. 9), (3) on a hook 2 (Figs. 3, 10, and 11A), and (4) behind the door bar (Fig. 13A, C).

**Fig.7:**
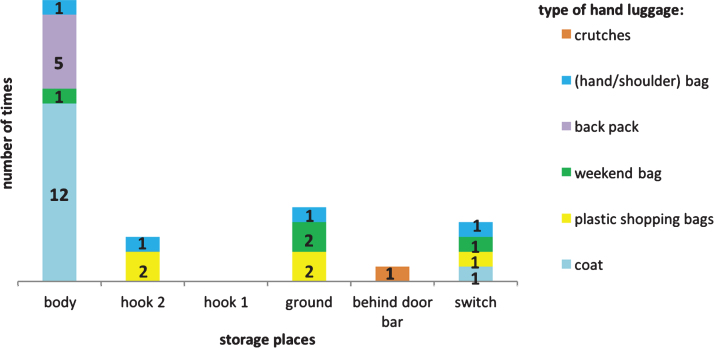
Places where the participants put their hand luggage.

**Fig.8:**
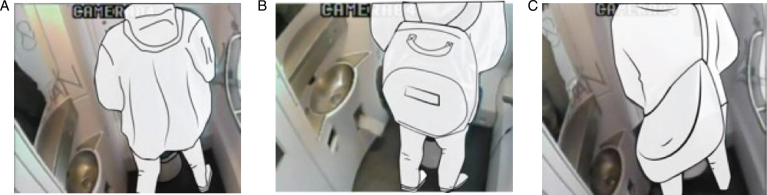
(A) Nr.16 keeps coat on. (B) Nr. 46 wears rucksack on the back. (C) Nr. 19 keeps weekend bag on the back.

**Fig.9:**
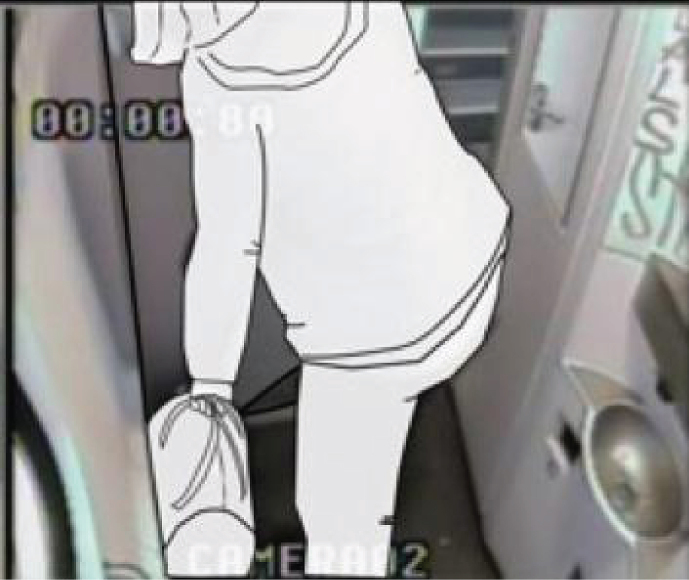
Nr. 21 drops weekend bag in corner on the ground.

**Fig.10:**
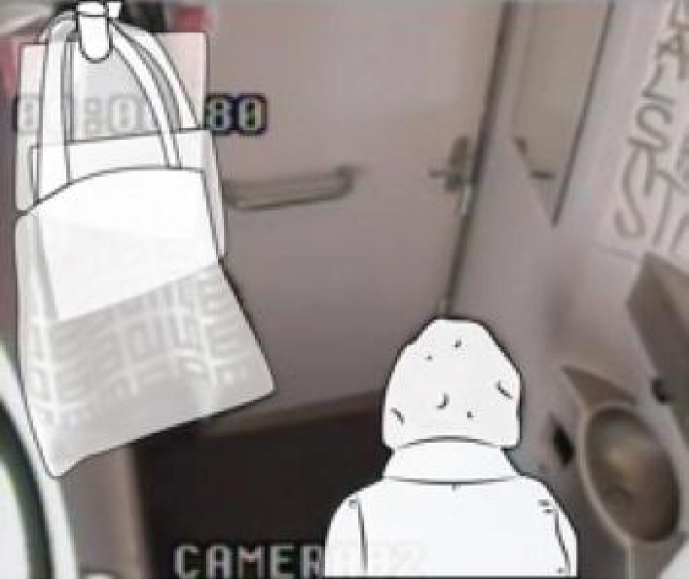
Nr.20: plastic bags and shoulder bag on hook.

**Fig.11:**
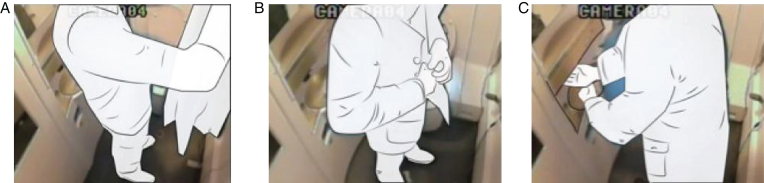
(A) Nr. 50 hangs up his coat on hook 2. (B) He buttons up his coat, halfway. (C) He keeps his coat on.

**Body**

**Fig.12:**
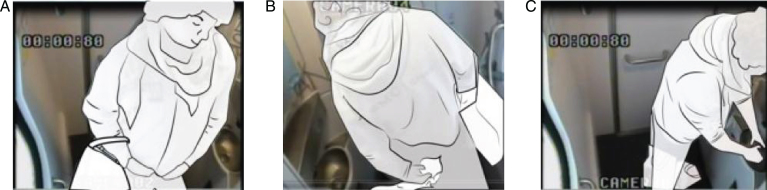
(A) Nr. 25 keeps plastic shopping bags around his wrist. (B) and he takes toilet paper. (C) Nr. 25 drops plastic shopping bags on the ground between his legs when washing the hands.

By ‘on their body’, we refer to the item of hand luggage being kept somewhere on the participants’ bodies; on their backs, around their wrists, and/or keeping their coat on. The body was the most commonly used storage space, (see Figs. 8, 11C, 12A, and B). In some cases this led to difficulties, for example in one observation, it was clear that the male participant was looking for somewhere to store his plastic shopping bags, but he could not find a suitable place. As a result, he put the bags around his wrist, and this hampered him in his performance in the train toilet (Nr. 25, Fig. 12A, B, C).

Of the 22 participants, 9 left their coats on their seat before they embarked on the observation.

The other participants kept their coats on, except (Nr.50) (referred to as switching), who directly hung up his coat on hook 2, but halfway through the observation he put on his coat again and washed his hands, Fig. 11A, B, C.

Five male participants kept their rucksacks on their back; none of the women had a rucksack. One male participant kept his weekend bag on the body, (Nr. 19), Fig. 8C. A female participant (Nr. 18) also did this initially, but as soon as she turned, she dropped the weekend bag in the corner (indicated as switching), Fig. 17.

**Floor**

In the corner on the floor was a popular place to drop the bags (5 times) in the large train toilet, see Figs. 2, 7, 9, and 17C. One female participant (Nr. 22) directly put her plastic shopping bags in the corner on the floor, and after a short while she dropped her handbag carefully above the shopping bags in the same corner. So, in the large train toilet, bags were dropped in the same spot, namely on the floor, in the corner, close to the door.

In the small train toilet, the floor was also used once to drop a bag (Nr. 30, Fig. 2, section 2.2).

**Hook 2**

In the toilets, an alternative hook (hook 2) was located at the far end of the support bar (see Figs. 3, 5, 10, 11A and 15); 140 mm lower than hook 1 at the height of 1700 mm from the floor. That was used to hang up bags by two participants, and once for a coat. The real purpose of this hook (contiguous with the support bar) was to hang up a triangle aid as a means for disabled users to transfer themselves to and from the toilet. Because of frequent misuse of this triangle aid, the NS decided to remove it from the supporting bar (information provided by Dutch Railways, NS).

**Switching**

During the observations, five participants switched their hand luggage from one place to another, see Figs. 11, 12 and 17. First, a male participant (Nr. 25) could not find a place to keep the three plastic shopping bags, which he then put around his wrist (noted as body) that hampered him in his movements. In between, before washing his hands, he dropped the bags on the ground between his legs, see Fig. 12. 

Another switch action was participant Nr. 30 who did the opposite in the small train toilet; he immediately dropped his bag on the ground and kept his coat on (Fig. 2). Subsequently, he picked up his handbag from the floor and kept it on his body while washing his hands.

In the third case, participant Nr. 50 hung up his coat on hook 2, and after sitting on the toilet and dealing with a stoma, he put on his coat, buttoned it up, before washing his hands (Fig. 11). Participant Nr. 18 kept the weekend bag on her back, but as soon as she turned her back to the wall, she placed the weekend bag in the corner, Fig. 17.

**Fig.13:**
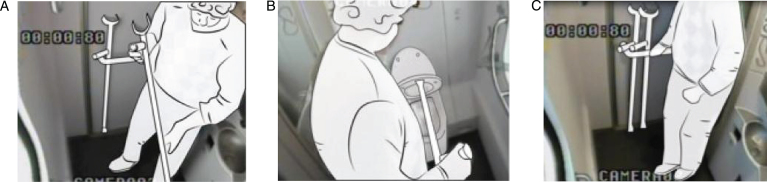
(A, B) Nr. 23 uses a crutch to raise the toilet seat. (C) Nr. 23 puts pair of crutches behind door bar.

Finally, participant Nr. 23 used crutches that he placed behind the door’s support bar, Fig. 13.

#### 3.2.2 Figures showing locations of hand luggage

**Body**

**Ground**

**Hook 2**

**Switching**

**Behind door bar**

## 4 Discussion

We discuss our main findings in the context of the aims of this article; to explore whether hand luggage, being a typical aspect of travellers’ journeys, is an issue when visiting the train toilet, to produce an overview of the involved hand luggage, and to discover where travellers leave their hand luggage in a train toilet environment.

We report that the 72 train travellers who replied to the two questions on hand luggage view the limited storage space in a train toilet as a problem, because of the lack of storage space, and that they are afraid of losing their seat. Consecutively, we looked at the different storage places of the 6 female and 16 male observations with respect to the differences between males and female participants, as gender is an important characteristic determining how people use a toilet (the main finding provided from an extensive questionnaire as part of the PhD thesis, to be published at a later date, see section 2.1).

### 4.1 Losing a seat when visiting the toilet carrying belongings

In the train compartment itself, different storage places are available, including hooks to hang up a coat, overhead luggage racks, and spaces behind the seat for luggage storage. Each train toilet door is fitted with a specially designed hook inside the door of at the height of 1840 mm from the floor, which for example, can be used to hang a coat.

We discovered that, in general, people prefer to keep a close eye on their personal belongings in a train environment, as it is a relatively anonymous public place. As a consequence, they do not ‘dare’ to leave their coats on their seats and prefer to take their personal belongings with them when visiting the train toilet. They reported both a lack of storage space and the risk of forfeiting their seat to another passenger.

### 4.2 Lack of storage space available in the train toilet

The only ‘designed’ storage place available in the current train toilet is hook 1, positioned high on the inside door. This hook was not used by any of the participants, while the alternative hook 2 positioned slightly lower, was used 4 times (Fig. 5).

### 4.3 Places where the participants kept their hand luggage

**Hook 1**

Although none of the participants used hook 1, three participants used hook 2, which is positioned slightly lower. Possible reasons given for not using hook 1 were firstly, it’s location at 1840 mm from the floor on the door (Fig. 5): this is seen as being too high; it is above average eye-height (1563 mm (F) and 1705 mm (M), and even above the average stature height of both men and women (1817 mm (M) and 1668 mm (F) [15]. This location is thus outside the participants’ reach comfort [15, 16].

**Hook 2**

The alternative hook 2 located 140 mm lower at 1700 mm from the floor, is within comfortable reach height.

However, a height of 1250 is within the comfort area for those who can only reach to a restricted height such as children, people with mobility difficulties or those who use a wheelchair [15, 16].

Secondly, this hook 2 is more recognisable as a hook, (Figs. 5 and 15) compared to the standard hook depicted in Fig. 16 [8 p147]. However, hook 1, (Figs. 4 and 5) with a flat surface was specially designed for this type of train, to reduce the chance that the hook could wound train passengers if they were thrown off balance by the train’s movement.

**Fig.14:**
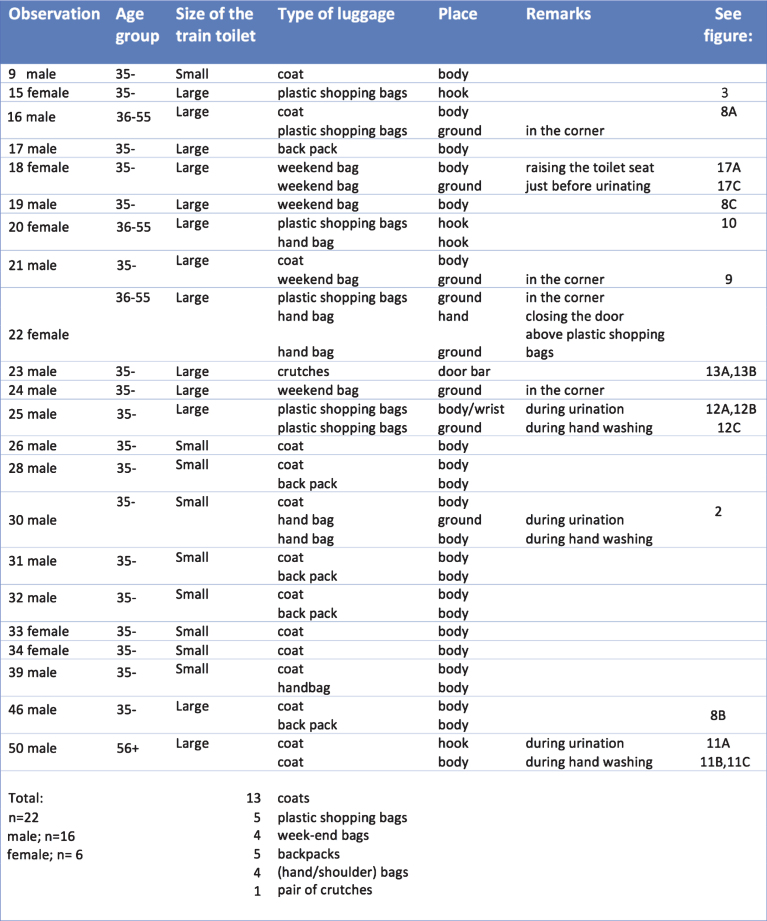
Table of hand luggage in the train toilet.

**Fig.15:**
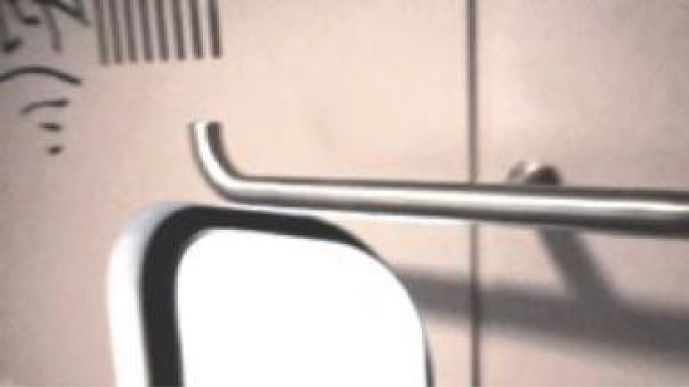
Hook 2: actually, mentioned for another purpose, positioned at the height of 1,700 mm from the floor.

**Fig.16:**
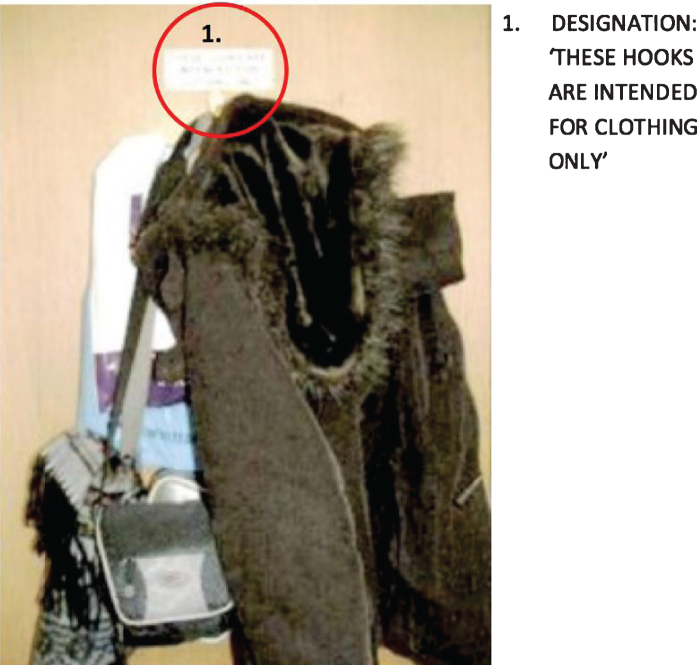
“Hook being used for coats and bags” [8 p147].

**Floor**

The large train toilet for disabled users (door width 765 mm) offered enough space to drop hand luggage on the ground; this occurred five times exactly on the same spot, close to the door. In contrast in the small train toilet, the floor space is too limited to put hand luggage, and the limited door width (544 mm) also hampers passengers with hand luggage.

However, when placing bags on the floor, the underside of the bag will become pick up bacteria [2, 17] which in turn can be transferred to more sensitive (body) locations [2].

**Body**

The participants’ favourite place to store their hand luggage was their own body, in particular, their coats and rucksacks (19 times, Figs. 7 and 14, (table hand luggage). This is logical as both coats and rucksacks are designed to be worn.

The other bags that participants kept on their bodies (a weekend bag, and shoulder bag) both had a suitable shoulder strap, although they could also have been hung up on a hook. Male participants, in particular, preferred to use their body as a practical alternative for the storage hook.

### 4.4 Differences between men and women

According to Rawls, 39% of the men carry an item with them [17], while Kira reports that every woman carries “at least a handbag” [18]. In our 22 observations, five men wore rucksacks, while none of the women did. Furthermore, two men and two women carried their own bag (Nr. 20, 22 and 30, 39) into the train toilet: the men kept their bags on their body, while the women did not (one hung the bag on hook 2, and the other carefully placed her personal bag above the plastic shopping bags on the floor.

It is thus likely, in the context of a public toilet, that the need for a storage place for women is more pressing than for men, as fewer men carry an item with them. We observed that it is easier for men to store hand luggage on their body, due to their position when using a toilet (face to the wall), so they have enough space left for hand luggage on their back, with a strap or rucksack. In contrast, women only have limited space on their bodies as they are in a hovering position or seated when using toilets with their back to the wall, (see Fig. 17 as an illustration).

### 4.5 Limitations of the study

**Fig.17:**
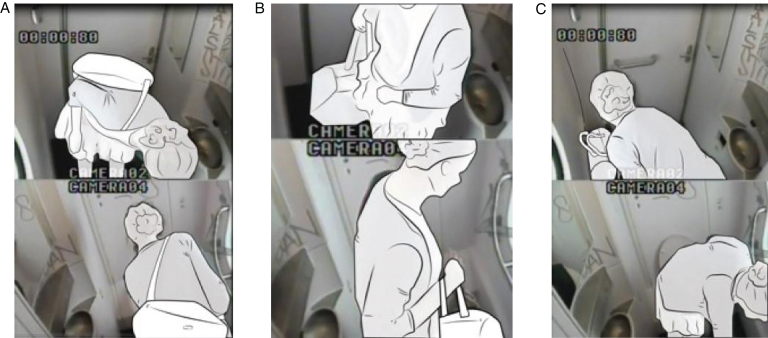
(A) Nr. 18 wears weekend bag on her back. (B, C) As soon as she turns, she drops the weekend bag on the ground in the corner.

#### 4.5.1 The questionnaire

The indirect way the questions on luggage were asked is likely to have influenced the number of respondents, as they were focused on the main research question about using train toilets.

Therefore, the total of 72 respondents commenting on personal belongings and hand luggage although sufficient for analysis, is however insufficient for drawing strong conclusions. A separate or more direct question on hand luggage would have probably increased the number of respondents.

#### 4.5.2 The observational research

Of the 22 participants who were observed, nine participants left their coat on their seat before they embarked on the observation; on a more typical train journey they may not have done this as they were seated together which may have resulted in a more relaxed situation that would be normal in a train. However, this may be realistic, as they may have left their coat on the seat to claim it; in the questionnaire, 22 respondents mentioned losing their seat as being an issue.

Although the sample size was too small to be conclusive, especially since we had noticeably less female observations (6) compared to male observations (16), the numbers were sufficient to give indications of use [19]. Furthermore, the sample is only partially representative for ordinary train toilet use, as the participants were mainly young, “able-bodied” students [8 p12]. In general, the older age groups were parents and friends of the students. The toddler and wheelchair user were not included in these luggage observations. The description of their toilet usage is in preparation. Nonetheless, the authors consider the information provided by the participants to be valuable with regard to what takes place in a moving train toilet.

## 5 Conclusion

Train travellers, especially females, need a facility in the train toilet where they can store their hand luggage. Travellers main concern is ‘good’ hygiene when storing their hand luggage in the toilet’s confined space, as it seems that they avoid dirty locations; most of our participants tried to store their hand luggage as far away from the (dirty) toilet bowl as possible, and the majority (14 of 22) did not place their luggage on the (dirty) floor.

Our observations show that male toilet users can use their backs as storage, while women may have a greater need for a hand luggage storage facility; they use the toilet while seated or in hovering position, with their backs to the wall, so they only have limited space on their backs for hand luggage.

The currently available facilities for coat and hand luggage storage in the train toilet remain underused. The storage hook is located on the door and is not used due to its height from the floor. Based on these findings, we recommend that the hook is positioned lower, at a maximum height of 1700 mm, which ensures that a coat will not touch the floor. Moreover, when adding a hook as a storage place, a second hook needs to be added for people with a shorter (reach) comfort area such as children, and people with mobility difficulties or those who use a wheelchair.

## 6 Recommendations for further (design) research

When designing adequate storage place in public toilets including train toilets, designers need to take both comfort and hygienic aspects into account. In the UK, a shelf is a requirement in disabled toilets [2, 12].

This appears to be a practical option [2, 12, 13] and Greed mentions it as an ideal solution, however further research is needed regarding hygienic aspects [2, 17].

Secondly, storage space on the travelers’ own bodies could be a practical alternative; this was noted several times in the observations. Designers of bags and coats need to take this into account.

Lastly, designers need to investigate how to provide an adequate storage place for other luggage items such as diapers, colostomy equipment, catheters, wheelchairs, walkers and strollers, as well as suitcases.

## Conflict of interest

None to report.
